# The emerging studies on mesenchymal progenitors in the long bone

**DOI:** 10.1186/s13578-023-01039-x

**Published:** 2023-06-10

**Authors:** Fangyuan Shen, Xiaobin Huang, Guangxu He, Yu Shi

**Affiliations:** 1 State Key Laboratory of Oral Diseases & National Clinical Research Center for Oral Diseases, West China Hospital of Stomatology, Sichuan University, Chengdu, China; 2 Department of Oral and Maxillofacial Surgery/Pharmacology, School of Dental Medicine, University of Pennsylvania, Philadelphia, PA, USA; 3 Department of Orthopedics, The Second Xiangya Hospital, Central South University, NO. 139 Middle Renmin Road, Changsha, Hunan China

**Keywords:** Mesenchymal progenitors, Bone development, Bone regeneration

## Abstract

Mesenchymal progenitors (MPs) are considered to play vital roles in bone development, growth, bone turnover, and repair. In recent years, benefiting from advanced approaches such as single-cell sequence, lineage tracing, flow cytometry, and transplantation, multiple MPs are identified and characterized in several locations of bone, including perichondrium, growth plate, periosteum, endosteum, trabecular bone, and stromal compartment. However, although great discoveries about skeletal stem cells (SSCs) and progenitors are present, it is still largely obscure how the varied landscape of MPs from different residing sites diversely contribute to the further differentiation of osteoblasts, osteocytes, chondrocytes, and other stromal cells in their respective destiny sites during development and regeneration. Here we discuss recent findings on MPs’ origin, differentiation, and maintenance during long bone development and homeostasis, providing clues and models of how the MPs contribute to bone development and repair.

## Introduction

**Fig. 1:**
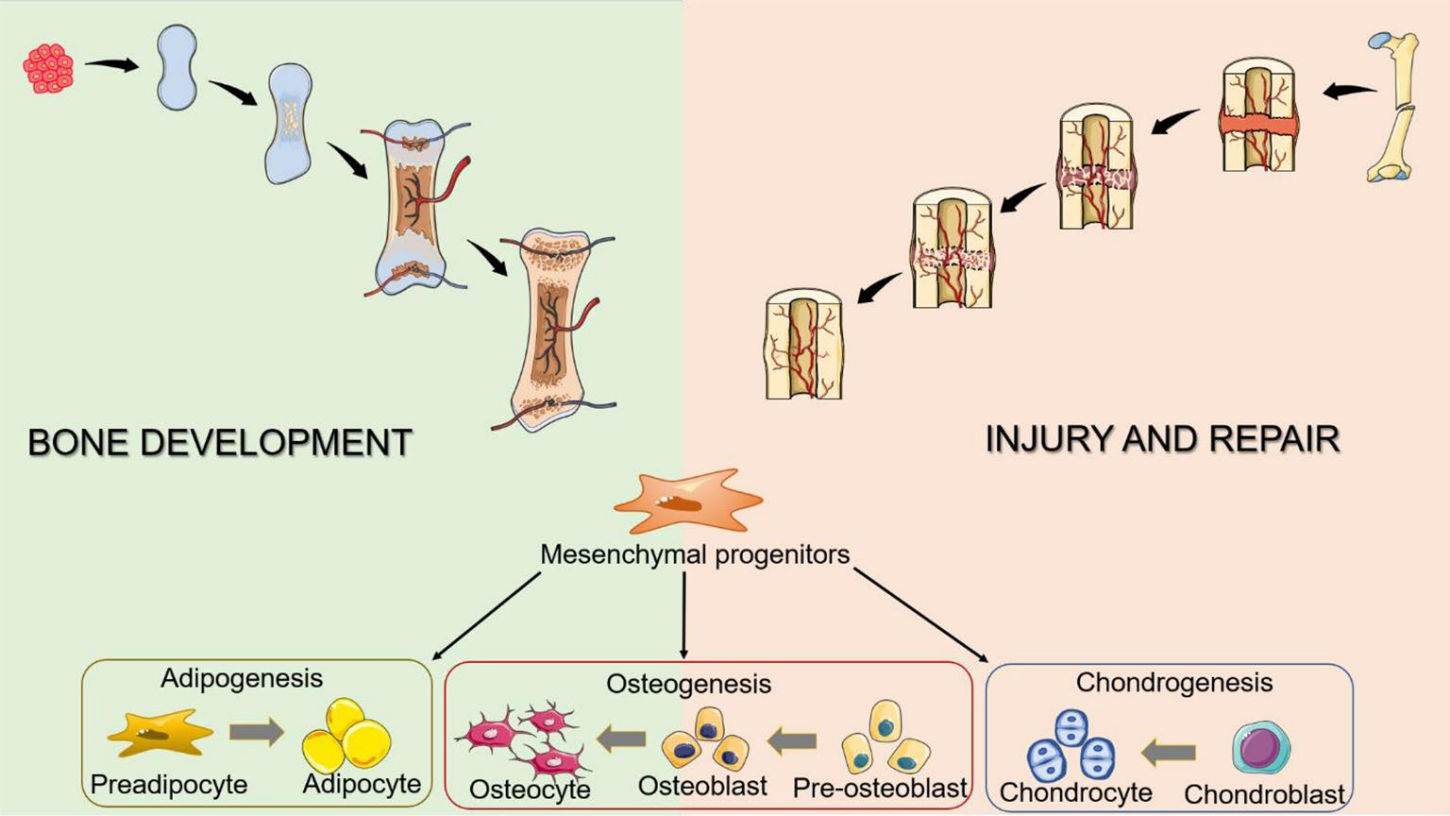
MPs are essential to skeletal development and repair

Bone and cartilage are the main tissues in the skeletal system, which are connected to muscle, tendon, and ligament to form an entire system that protects the inside organs, allows body movement, secretes hormones to regulate the whole body’s metabolism, and stores hematopoietic stem cells (HSC) in the bone marrow. There coexist distinct cell types in bone tissue including osteoblast, osteocyte, chondrocyte, osteoclast, adipocyte, and epithelium, as well as MPs, and more determined precursors. The deliberate coordination of the coexisted multiple cells is essential to skeletal development, maintenance, and repair (Fig. 1). Compared with the well-elucidated hematopoietic system, the stem/progenitor cell regulation in the skeletal system remains relatively unexplored.

The condensation of mesenchymal cells is the first event of skeletal development and regeneration, followed by endochondral ossification or intramembranous ossification. The condensed mesenchymal cells are the initial source of MPs. Generally, the gold standard to define MPs is their self-renew capability and multilineage potential, especially the capability to differentiate into adipocytes, osteoblasts, marrow stromal cells, chondrocytes when cultured in vitro, and ossicles forming upon heterotopic transplantation into immunodeficient mice [1–3]. However, the in vivo identities of MPs remain largely understudied. In 2015, Longaker's team for the first time identified stem cells that only formed the osteogenic lineage (osteoblasts, chondrocytes, and stromal cells) in rapidly dividing cell populations in mouse bones and named them SSCs [4]. Since then, the conception of SSCs derived from MPs is commonly accepted. MPs initiate bone formation by generating a series of progressively fate-limited precursors. Then the precursors directly provide the source of functional cells including osteoblast, osteocyte, chondrocyte, and adipocyte. Therefore, condensed mesenchymal cells could give rise to different descendants in order of MPs, SSCs, precursors, and different final cell types source to feed the stage and tissue-specific cell requirement. The complete network of the skeletal system is built by the cooperation of multiple stem cells, precursors, and final differentiated cells.

Due to the high heterogeneity and lack of specific markers, it is difficult to define MP populations which is at the apical point of the differentiation hierarchy and their respective functions to distinguish from their tissue origin cells. Many efforts have been put into characterizing and isolating this kind of SSCs during bone development, growth, and repair. In recent years, multiple new markers of MPs and SSCs are identified, and accordingly, new progenitor populations are explored [4–8]. For example, bone marrow stromal cells were widely labeled by Thy1, Eng, Nt5e, and CD44, with the variable ability of colony-formation and multilineage differentiation to generate osteoclast, chondrocyte, and adipocyte. In addition, Ngfr and Mcam could likewise mark stromal cells in bone marrow [9, 10]. Furthermore, MCAM^+^ subendothelial cells from human bone marrow stroma also recreated hematopoiesis-supportive ossicles in a mouse [3]. Nevertheless, some of these markers might just define subpopulations rather than unique populations and cannot distinguish from the original cells. How the MPs differentiate into tissue-specific and stage-specific precursors, how the precursors generate terminally differentiated cells, and what is their origin and travel ways during differentiation are still elusive. The regulatory and genealogical hierarchy of MPs remains largely unexplored.

In this review, we will address these questions: (1) what are the locations of varied MPs? (2)when and how do the MPs move to the locations? (3) what are the markers combinations for distinguishing different MPs populations? (4) Which MPs response to bone injury, and are responsible for repair and remodeling? To simplify the whole process, we take the mice’s long bone formation as an example, but the mechanisms could likewise apply to other bones or species.

## Origin, development, and aging of MPs

MPs are generated and evolved during the processes of bone development. The nature and state of stem cells may be varied from stage to stage, and different between site by site. In bone tissue, in addition to bone marrow as the main location of MPs and corresponding precursors, other sites such as growth plate, periosteum, endosteum and trabeculae could also be niches for MPs in fetal and postnatal stages. Here, we elucidate the evolution of the discrepant MP populations based on the processes of bone formation, development, and aging (Tables 1 and 2).

**Table 1:** Characterization of embryonic and fetal derived mesenchymal progenitors

Development stage	Location	Cell name in reference	Marker	References
Mesenchymal condensation	Limb bud	Limb mesenchymal cells	Prx1^+^	[12]
Mesenchymal condensation	Limb bud	Musculoskeletal precursors	Hoxd13^+^/Tbx13^+^	[13]
Mesenchymal condensation	Limb bud	Osteo-chondrogenic progenitors	PRRX1^+^ PDGFRA^+^SOX9^low^	[14]
Cartilage template	The core of the limb bud mesenchyme		Sox9^+^	[15]
Cartilage template/POC formation	The growth cartilage and perichondrium		Col2^+^	[18]
Cartilage template/POC formation	The growth cartilage and perichondrium		Sp7^+^	[18]
Perichondrium formation/POC formation	The innermost part of the perichondrium		Nes^+^	[21]
Perichondrium formation	The outer layer of perichondrium		Hes1^+^	[22]
Perichondrium formation	The outer layer of perichondrium		Dlx5^+^	[22]
Perichondrium formation	Perichondrium		Pthlh^+^	[23]
Fetal growth plate development	The second half of pre-hypertrophic zone and first half of hypertrophic zone in the fetal growth plate	Human skeletal stem cells	PDPN^+^CD146^−^ CD73^+^CD164^+^	[5]
Fetal growth plate development	Chondro-osseous junction		Col10a1^+^	[25]

**Table 2:** Characterization of postnatal long bone derived mesenchymal progenitors

Development stage	Location	Cell name in reference	Marker	References
From embryonic to postnatal	Periosteum	Periosteal stem cell	CTSK^+^THY^−^6C3^−^CD49^low^CD51^low^ CD200^+^CD105^−^	[16, 52]
From embryonic to adult	Primary spongiosa		Sp7^+^	[20, 51, 81, 82]
From the neonatal to the growing stage	The border region of growth plate		Axin2^+^	[4, 51]
Newborn	The top of the resting zone of the growth plate	Long-term skeletal stem cells	FoxA2^+^Col10^−^	[50, 52]
Postnatal	Growth plate	Mouse skeletal stem cells	CD45^−^Ter119^−^Tie2^−^AlphaV^+^Thy^−^6C3^−^CD105^−^CD200^+^	[4, 63]
Postnatal	The borderline and the resting zone of the growth plate		Pthlh^+^	[50]
Postnatal	Bone marrow perivascular	Cxcl12 abundant reticular cells	Cxcl12^+^	[65]
Postnatal	The chondro-osseous junction immediately beneath the growth plate		PDGFRβ^+^	[77]
Postnatal	Primary spongiosa		αSMA^+^	[39, 79]
Postnatal	Immediately outside of innermost Col1a1 + cells of the periosteum	Osteochondral progenitor cells	Prrx1^+^	[39, 85, 86]
Young	The chondro-osseous junction immediately beneath the growth plate	Metaphyseal mesenchymal progenitors	Gli1^+^	[20, 79, 81, 82]
Young	Periosteum		Nestin^+^PDGFRα^+^	[7, 88]
Adult	Periosteum		LepR^+^	[85, 86, 88]
Adult	Surrounding sinusoids and arterioles in bone marrow	Bone marrow stromal cells	LepR^+^	[63, 88]
Adult	The cambial and fibrous layers of the periosteum		Gli1^+^	[88, 109]
Adult	Primary spongiosa and osse-chondro junction	Osteochondroreticular stem cells	Grem1^+^	[7, 16]
Adult	Perivascular and distributed throughout the bone marrow		LepR^+^Adipoq^+^	[109]

### Putative source of MPs in embryonic and fetal stage

#### Mesenchymal condensation of early embryonic development

Mesenchymal condensation is a prerequisite process for the following endochondral bone development. Mesenchymal cells that form limb bud are derived from the lateral plate mesoderm, and they cluster tightly together to build a primordial structure that is the frame for future bone formation under the precise regulation of various pathways including Eph-ephrin, Sonic Hedgehog, Notch, Bone Morphogenetic Protein (BMP), and Fibroblast Growth Factor (FGF) [11]. These cells are typically marked by *Prrx1* (*Prx1*), which is expressed as an essential transcription factor mediating undifferentiated mesenchyme to form pre-skeletal condensations. By lineage tracing, the *Prxx1* could mark all limb mesenchymal cells [12]. Therefore, the condensed mesenchymal cells serve as the common original source of MPs, various precursors, osteoblasts, osteocytes, chondrocytes, perichondrial cells, adipocytes, and other stromal cells. Single-cell RNA-sequencing of mouse hind limb (E11.5) and human embryonic limb buds (5 weeks post conception) revealed significant cell type heterogeneity corresponding to various functional cells in the later life [13, 14], suggesting that cellular diversity emerged even at this relatively homogeneous stage. Among these heterogeneous cell populations, mice musculoskeletal precursors expressed *Hoxd13* or *Tbx13* [13]; meanwhile, human limb bud mesenchymal cells expressed *PRRX1*, and osteo-chondrogenic progenitors were labeled by PRRX1^+^PDGFRA^+^SOX9^low^ [14], all of which could be primogenitors with potential for MPs generation.

#### Chondrocyte differentiation of fetal stage

After mesenchymal condensation, the center mesenchymal cells differentiate into chondrocytes while several layers of the periphery mesenchymal cells become perichondrial cells and eventually establish perichondrium. Sox9 is the earliest known transcription factor to regulate the expression of major cartilage matrix genes *Col2a1*(*Col2*) and *Aggrecan (Acan)* which produce collagens and proteoglycans respectively, further establishing cartilaginous extracellular matrix [15]. Cells expressing *Sox9*, *Col2a1*, *Acan*, and *Fgfr3* generate chondrocytes, followed by hypertrophic chondrocytes expressing *Col10a1*. Early chondrocytes expressing *Sox9* and *Col2a1* function as the main origin of stromal cells and osteoblasts during endochondral bone formation. Lineage tracing experiment utilizing Sox9-creERt transgenic mice indicated that earlier Sox9^+^ cells could be chased into stromal cells besides growth plate chondrocytes. These stromal cells were divided into three groups: (1) cells immediately adjacent to the growth plate; (2) perichondrium; (3) immediately below the bone lining cells of trabecular bone, endosteum and periosteum [16]. Intriguingly, Sox9^+^ descendant cells could also be adipocytes, besides osteoblasts and stromal cells [17]. Col2^+^ cells show a similar fate map with that of *Sox9* positive ones. A majority of osteoblasts, Cxcl12^+^ abundant reticular cells (CAR cells), and bone marrow mesenchymal progenitors (BMMPs) were all derived from Col2^+^ cells [18]. Paradoxically, some researchers believed that Sox9-cre have already marked all chondrocytes and osteoblasts [18]. Therefore, we still need to further clarify the relationship between Sox9^+^ and Col2^+^ MPs: whether these two markers define the same MP population, or whether there are MP subsets that express both markers. Additionally, after the progressive differentiation of mesenchymal cells into chondrocytes, it appears that a proportion of MPs in mesenchymal condensation areas do not undergo chondrogenic differentiation and maintain a stem cell-like state, except for the central chondrocytes that will undergo hypertrophy and the future periosteal cells located in the periphery [18].

#### The formation of perichondrium in fetal bone

Around the same time as chondrogenic differentiation, the perichondrium is formed and comprises two layers: outer fibrous layer and inner chondrogenic layer, surrounding the condensation. At E12.5, *Col2* labels proliferative perichondrium cells. Furthermore, at E14.5, when chondrocytes hypertrophy in the center of cartilage template, one group of perichondrium inner cells start differentiation and express *Osx* (*Sp7*), a key transcription factor for osteogenic differentiation. *Col1*^+^ cells appear in the perichondrium at this time and *Runx2* is required for this process since *Runx2*-deficient bone anlage leads to the absence of Col1-GFP^+^ cells at E14.5 [18]. Both Sp7^+^ and Col1^+^ cells are derived from Col2^+^ cells and located inner portions of the perichondrium. However, only *Sp7*-expressing perichondrial cells intrude into the hypertrophic center with vascular system and transiently contribute to both bone marrow osteoblasts and stromal cells that subsequently support primary ossification center (POC) formation [19, 20]. Besides, *Nes*-GFP which marked endothelial and non-endothelial *Nes*^+^ cells in the perichondrium but not in mesenchymal condensation, were also the descendants of Col2^+^ cells in the fetal perichondrial cell lineage. At E13.5–14.5, Nes^+^ cells mostly emerged and dominated the innermost part of the perichondrium adjoining the nascent hypertrophic chondrocytes and vasculature. In the perichondrium, MPs become Nes^+^ cells of the osteoblast lineage and subsequently Sp7^+^ preosteoblasts, both in an Indian Hedgehog/Ihh and Runx2-dependent manner [21].

Hes1, a Notch signaling transcription factor, is abundantly expressed in outer layer of perichondrium. By using Hes1-creERt mice, one study exhibited that Hes1^+^ cells generated osteoblasts and stromal cells more persistently and to a greater extent than Sp7^+^ cells in the fetal perichondrium [2]. Importantly, Hes1^+^ cells serve as an important MPs source in limb bud, which surround but do not overlap with Sox9^+^ pre-cartilaginous condensations at E10.5-E13.5, and provide precursors for most of perichondrial cells and a small population of Sox9^+^ chondrocytes in cartilage template [22]. Unlike Hes1 which label skeletal muscle cells and endothelial cells besides osteo- and chondro-lineage, Dlx5 is a perichondrial cell-specific marker that is highly active in the outer layers of the fetal perichondrium, and Dlx5^+^ cells continuously provide to a significant proportion of osteoblasts and stromal cells in the bone marrow [22]. In addition, Parathyroid hormone-related protein (PTHrP/Pthlh) positive chondrocytes in the periarticular areas of perichondrium during fetal stage have the potential to act as progenitors as well. Lineage‐tracing experiments by Pthlh‐creER; R26RtdTomato mice indicated that both Pthlh‐E14.5 and Pthlh‐E17.5 cells at P9 became metaphyseal endosteal cells and cells on articular surface, suggesting that *Pthrp* marked precursors of stromal cells and articular chondrocytes in the perichondrium [23].

Taken together, both cartilage template and perichondrium are MPs niches and highly coordinate the endochondral bone formation by manipulate MPs’ differentiation.

#### MPs in growth plate development of fetal stage

The formation of cartilage primordia triggers chondroblasts from diaphyseal and metaphyseal mature to construct growth plates in which prehypertrophic chondrocytes, hypertrophic chondrocytes and terminal hypertrophic chondrocytes are arranged from proximal to distal. MPs first differentiate in columnar chondrocytes before exiting the cell cycle, subsequently stop proliferating and sequentially activate PTHLH receptor, *Ihh* and *Col10*, after which chondrocytes become hypertrophy. Eventually the hypertrophic chondrocytes go through apoptosis or transdifferentiation, paving the way for the transition from cartilaginous anlage to bone.

In 2018, Longarker’s lab has identified PDPN^+^CD146^−^CD73^+^CD164^+^ marked cells as a group of multipotent human skeletal stem cells (hSSCs) with self-renewal ability, mainly located in the second half of pre-hypertrophic zone and first half of hypertrophic zone in the human fetal growth plate. Unlike mesenchymal stem cells, hSSCs can only produce progenitors of stroma, bone, and cartilage, but cannot produce adipogenic lineage cells [5]. Besides the Sp7-expressing cells from perichondrium, the Sp7-expressing prehypertrophic chondrocyte could also transdifferentiate into stromal cells and osteoblasts in the bone marrow [18]. Many evidence demonstrated that some terminal chondrocytes could transdifferentiate into osteogenic lineage cells rather than being replaced by osteoclasts [24–26]. The expression of Sox9 is downregulated in prehypertrophic chondrocytes, while *Runx2* is upregulated to promote chondrocyte hypertrophy and terminal differentiation [24], as well as osteogenesis. During osteogenesis, RUNX2 is a transcription factor necessary for chondrocyte maturation and osteoblast differentiation, and Sp7 functions as a downstream modulator of *Runx2* gene, which together with the canonical Wnt signaling pathway induces cells to osteoblast. *Runx2* and *Sp7*, markers of the osteoblast lineage, are found to be expressed in prehypertrophic chondrocytes and hypertrophic chondrocytes as well, which mean these cells share some property with osteoblast lineage cells. This provides a clue that the chondrocytes change into osteoblasts by two possibilities: (1) the chondrocytes directly transfer into osteoblasts or the precursors; (2) chondrocytes undergo dedifferentiation into mesenchymal progenitors which then further differentiate into osteoblasts. Lineage tracing provides robust evidence for the transdifferentiation of hypertrophic chondrocytes into osteoblasts, revealing a novel cell fate for chondrocytes during development in addition to apoptosis and autophagy. Col10a1 as a typical hypertrophic chondrocyte marker, Col10a1-creER lineage trancing indicated that Col10a1^+^ cells overlapped with Sp7^+^ and Col1^+^ cells existing at the chondro-osseous junction directly underlying the growth plate, demonstrating the potential for osteogenic commitment of hypertrophic chondrocytes during bone development [25–27]. BMP and Wnt/β-Catenin are pivotal signaling pathways for chondrocyte transdifferentiation [28]. It was shown that there are bone regional differences in the regulation of chondrocyte transdifferentiation by BMP during development [29]. Specific knockdown of Bmpr1a in Acan-CreERT2 cells resulted in a complete arrest of chondrocyte transdifferentiation in the growth plate, leading to impaired metaphyseal development, and thus BMP showed a pro-transdifferentiation effect in the metaphysis. In contrast, an increase in chondrocyte-derived osteoblasts in the epiphysis led to the malformed expansion of the epiphysis, suggesting a transdifferentiation-inhibiting effect of BMP in the epiphysis [29]. Transdifferentiation of chondrocytes in the long bones of transgenic mice with specific abrogation of β-catenin activity in hypertrophic chondrocytes was almost completely blocked, and cartilage-derived osteoblasts beneath the chondro-osseous junction were dramatically reduced [30]. In addition to signaling pathways, Runx2 and Sox9 are now known to be essential transcription factors of chondrocyte transdifferentiation [29]. Elimination of Runx2 in Col10a1-Cre-labeled hypertrophic chondrocytes increases apoptosis and interrupts transdifferentiation, causing the impaired formation of primary spongiosa [31], whereas sustained expression of Sox9 in chondrocytes inhibits osteogenic transdifferentiation and downregulation of Sox9 promotes osteogenesis-related gene expression in hypertrophic chondrocytes and induces transdifferentiation into osteogenic lineage cells [32, 33].

#### POC formation and the source of MPs in fetal bone marrow cavity

Except for the growth plate cartilage, most chondrocytes and the secreted matrix in the cartilage anlagen have been replaced by vascularized bone structure and bone marrow, right after POC formation. The centered matrix is degraded, and hypertrophic chondrocytes undergo apoptosis in the presence of matrix metalloproteinase-9 expressed in chondroclasts [34], and followed by cartilage angiogenesis. Blood vessels invade into the lacuna of cartilage matrix from perichondrium under the mediation of vascular endothelial growth factor (VEGF) expressed by the hypertrophic chondrocyte. The blockade of VEGF may lead MPs to facilitate chondrogenesis at the cost of osteogenesis [4]. The MPs are recruited into the nascent bone marrow cavity, and then osteogenic precursors differentiate into osteoblasts to establish the POC and develop the trabecular bone [19]. We reviewed several sources of MPs in nascent bone marrow as follows.

1. Adjacent perichondrium. Most of the osteogenic progenitors in the formation of nascent bone marrow originate from the perichondrium. At E14.5–E15.5, POC begin to form as vessels invading into cartilage anlagen, while Col2^+^, Nes^+^ and Sp7^+^ cells located in perichondrium enter the nascent marrow cavity with blood vessels, referred as precursors of osteoblasts and stromal cells [18, 19, 21]. Moreover, Col2^+^ cells are more ancestral than Sp7^+^ and Nes^+^ cells. The lineage tracing data showed that the perichondrial Col2^+^ cells as early as E11.5 could serve as the source of osteoblasts in the bone marrow cavity and Col2^+^ cells labeled at E13.5 continuously generated descendants which could be traced in the growth cartilage, the perichondrium at P0 and the secondary ossification center in the epiphyseal at P21. In contrast, Sp7^+^ cells appeared in the perichondrium around the bone collar at E13.5 and its descendants were confined to bone marrow region at E16.6 and P0, ultimately disappearing in perichondrium [18, 19]. Descended from Col2^+^ cells in perichondrium, non-endothelial Nes^+^ cells of the osteoblast lineage proliferate in the perichondrium and interact closely with endothelial Nes^+^ cells, partly as Sp7^+^ osteoblast precursors [21]. However, it is still not clear how the stem cells and precursors communicate with the vessel for migration.
2. Hypertrophic zone of growth plate. Osteoblasts can be transdifferentiated from chondrocytes, and chondrocytes from the perichondrium prior to primary ossification as well as from the growth plate are capable of transdifferentiating and becoming osteoblasts. Based on lineage tracing experiment, a number of evidences illustrated that hypertrophic chondrocytes could transdifferentiated into osteoblasts right under the hypertrophic zone [25, 26]. Approximately more than half of the mature osteoblasts in metaphyseal trabeculae and endosteal surfaces of one-month-old mice were derived from Col10a1-expressing hypertrophic chondrocytes [26]. Aforementioned *Col2*, *Sox9* and *Acan* also mark stromal cells, some of which stand for SSCs in the newly established marrow. Therefore, chondrocytes are able to dedifferentiate into SSCs as a pool for further differentiation into osteoblasts.
3. From other tissue. There might be some MPs along with HSCs migrated from adjacent tissues, since the vasculature is a dynamic system in the body to connect all the tissue and organs [35]. MPs express a wide range of chemokine receptors, such as CC chemokine receptors and CXC chemokine receptors, that drive MPs to migrate into adjacent tissues [36]. Indeed, mouse bone marrow MPs are composed of the combination of neural crest-derived [37] and mesoderm-derived cells [38, 39], whereas MPs are readily detected in the human fetal circulation [40]. In particular, the vessel wall contains reserve of progenitor cells. Pericyte/perivascular cells share characteristics with MPs, and exhibit potential for adipogenesis, osteogenesis, and chondrogenesis. Crisan el at. revealed that no differences in cell morphology, cell markers, proliferation kinetics, and differentiation potential, between purified and passaged perivascular cells from different human organs and bone marrow-derived MSCs, either in vitro or in vivo [41].

MPs in bone marrow are highly heterogeneous from the time of formation, due to the diverse cellular origins. Even after birth, the traces of origin diversity can still be found in the BMMPs by means of lineage tracing and single-cell RNA sequencing among others [42, 43]. Therefore, it is reasonable to argue that the foundation for the complex bone marrow microenvironment in adult has been laid at this time.

### Postnatal MPs support bone growth and homeostasis maintenance

#### Secondary ossification center (SOC) postnatal formation

Unlike POC formation which occurs in embryonic stage, the formation of SOC is a postnatal event in the epiphyseal regions of long bones. Similar to POC formation, firstly the chondrocytes at the centers of the epiphyseal cartilage undergo hypertrophy and apoptosis. MPs enter the epiphyseal cartilage center along the vessels and differentiate into osteogenic lineage cell, thereby establishing the SOC. The cartilage template is replaced by the new formed bone, dividing the pre-existing cartilage into articular cartilage and growth plate [44].

As aforementioned, osteogenic lineage cells in ossification center are mainly derived from MPs which are arise from vascular invasion and the transdifferentiation of chondrocytes. With the vascularization in epiphysis cartilage, cells expressing mesenchymal lineage marker CD90 and CD105 are appeared in SOC and co-localize with CD34^+^ immature endothelial cells of the newly sprouting vessels [45]. Osx in chondrocytes, as an essential factor for hypertrophic chondrocytes transdifferentiating into osteoblasts, is crucial for the formation of SOC in epiphyses. In Osx-knockout mice, the expression of chondrocyte hypertrophic marker metalloproteinase 13 (*Mmp13*) and *Col10* were downregulated and the formation of hypertrophic chondrocytes and osteoblasts were significantly delayed, resulting in impaired SOC formation which causing the compromised cartilage-to-bone conversion [46].

#### Composition and localization of MPs in the postnatal growth plate

**Fig. 2:**
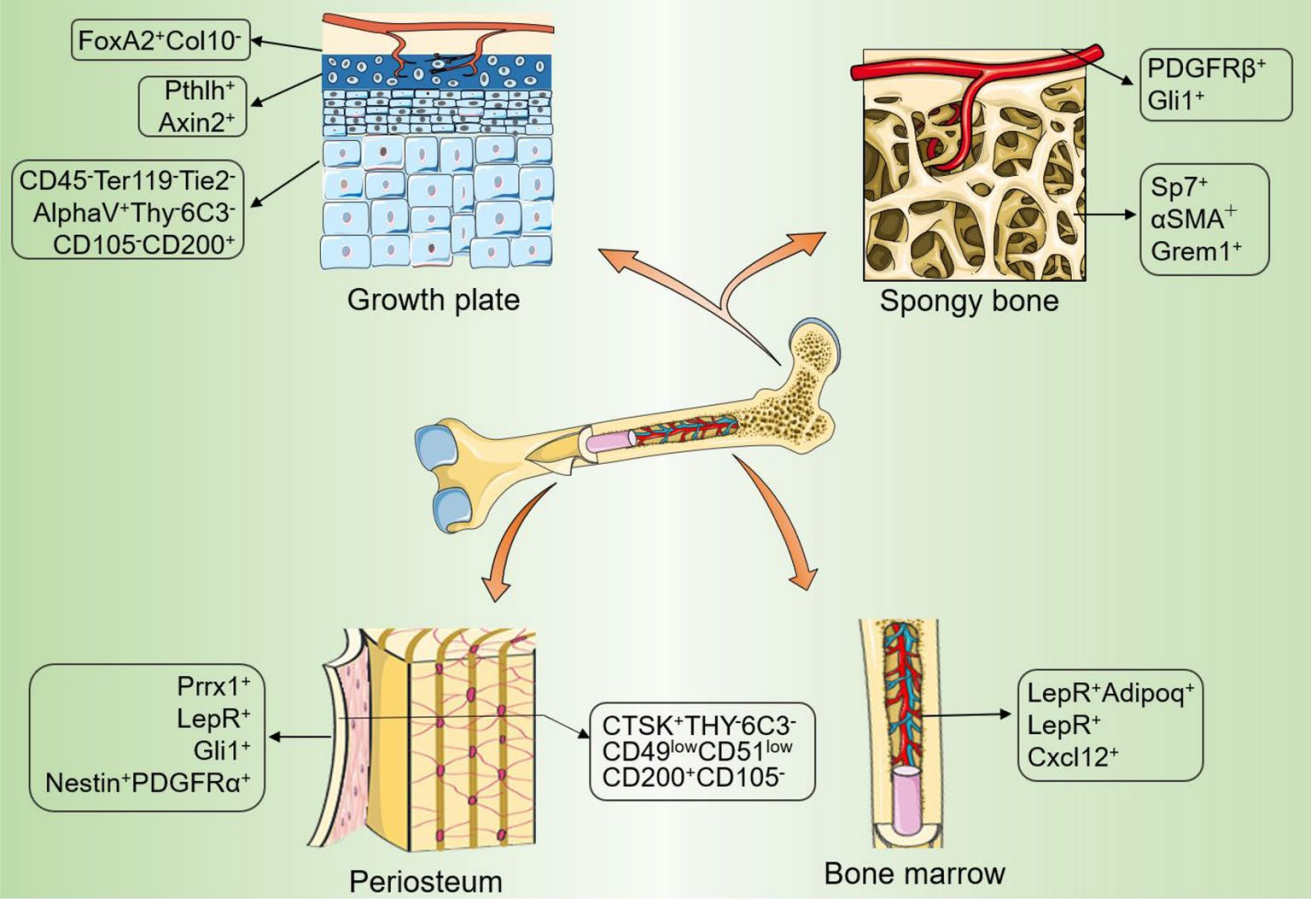
Mesenchymal progenitors residing in long bones

Since the perichondrium surrounding diaphysis eventually transforms into periosteum and cortical bone, the postnatal growth plate harbouring osteo-chondrogenic cells will be a main force for trabecular bone formation. The postnatal growth plate is divided into four parts starting from epiphysis to the diaphysis, namely the resting zone, the proliferative zone, the prehypertrophic zone, and the hypertrophic zone (Fig. 2).

Bone elongation is dictated by the expansion and hypertrophy of chondrocytes in epiphyseal cartilage [47, 48]. During fetal–neonatal in mice, chondroprogenitors are gradually depleted along with longitudinal skeletal growth. While later in life, due to the formation of SOC which alters the local microenvironment, a stem cell niche is progressively created in the growth plate. PTHrP/PTHLH, a protein member of the parathyroid hormone family, was discovered as a marker for SSCs residing at the borderline chondrocytes of growth plate in recent years. Borderline chondrocytes are located underneath the perichondrium after the formation of the cartilage primordia and ultimately on the border of the lateral sides of the postnatal growth plate as development progresses [49]. Lineage tracing experiments of Pthlh-creER mouse had indicated that a small group of borderline chondrocytes which were marked by *Pthlh* at P0 escaped from hypertrophy and entered the marrow to differentiate into osteoblasts and Cxcl12-CAR cells in trabecular bone. Interestingly, most of these cells disappear in the marrow after 9.5 weeks of chasing, suggesting that Pthlh^+^ borderline chondrocytes act as progenitors cells of stromal cells and osteoblasts which transiently persisted in the bone marrow for rapidly growing bones [50]. Chondrocytes in the early postnatal growth plate, labelled by *Sox9*, *Col2*, and *Acan* progressively contribute to osteoblasts, chondrocytes, and stromal cells for over a year, unambiguously demonstrating that these marked cells may represent MPs of skeletal development through dedifferentiation or transdifferentiation [18, 26]. Wnt-responsive cells labelled by *Axin2* which reside in the border areas of growth plate adjacent to the Ranvier’s groove, are another source of growth plate chondroprogenitors. Axin2^+^ cells contribute progressively to chondrocytes inside the growth plate from the neonatal to the growing stage, and are involved in the growth of growth plate in the lateral direction [51].

Since the postnatal growth plate is the middle of the sandwich composed by SOC, growth plate and POC, the highly vascularized marrow microenvironment in the SOC and POC could affect the dedifferentiation and transdifferentiation of chondrocytes in the resting zone and hypertrophic zone, or MPs from SOC and POC might directly migrate to growth plate. Adjacent to the SOC is the resting zone, lying at the apex layer of the growth plate. Cells in the resting zone are considered with low proliferative capacity and continuously self-renewal, producing progenies for the proliferative zone right below, which further become hypertrophic chondrocytes. Subsequently, hypertrophic chondrocytes transdifferentiate into osteoblasts and bone marrow stromal cells. IHH, PTHLH, FGF, BMP, NOTCH and WNT signaling collaboratively control the proliferation and differentiation of chondrocytes in growth plate. Especially, the classical negative feedback loop between IHH and PTHLH are involved in the regulation of growth plate dynamics in important way [46]. During adolescence when SOCs are formed, under the influence of IHH and mammalian target of rapamycin complex 1 (mTORC1) signaling pathways, chondroprogenitors in the resting zone start to express stem cell markers (CD73 and CD49e) and acquire the ability to self-renew and trilineage differentiation [46]. A similar stem cell subpopulation is the FoxA2^+^Col10^−^ population inhabiting the top of the growth plate resting zone, adjacent to the SOC. FoxA2^+^ cells show osteogenic and chondrogenic potential from P0-P28, but after P28 their fate is limited to chondrogenesis. And they are mainly retained in the growth plate as long-term skeletal stem cells, which are required for growth plate damage repair and turnover [52]. Furthermore, the postnatal resting zone houses another MPs population labelled by *Pthlh* which can become columnar chondrocytes for a long term, then further undergo hypertrophy and finally turn into marrow stromal cells and osteoblasts in epiphyseal trabecular. Due to its restricted lineage commitment comparable with MPs and the expression of a set of skeletal stem/progenitor cell markers, Pthlh^+^ chondrocytes in resting zone are assigned as MPs [50]. The interaction of Pthlh signaling from the resting zone and Ihh signaling in the hypertrophic zone seems to be important to maintain the MPs and their niches [50]. Moreover, it is fundamentally different between the Pthlh^+^ borderline chondrocytes which cannot self-renew and Pthlh^+^ resting chondrocytes that are enabled to perform long-term self-renewal within the growth plate resting zone. The specific mechanisms that cause Pthlh^+^ chondrocytes at the periphery differ from those of center are unclear and require further research.

#### Diversity of cell types in the postnatal bone marrow cavity

During adulthood, both growth plate activity and bone growth slow down and gradually stop, but bones still need to be constantly turnover and maintain their functions throughout the whole lifespan. The bone marrow, as a primary site of life-long hematopoiesis, possesses a specialized microenvironment which contains vascular system, hematopoietic lineage cells, and heterogeneous mesenchymal cells that crosstalk with each other to maintain the proper function of HSCs and MPs. The bone marrow niches specifically contains several subtypes of MP (Fig. 2), mostly associated with arterioles or sinusoidal blood vessels and located in the perivascular space [53]. Although, reporter genes have been extensively studied to purify osteo-lineage stem/progenitor cell populations, bone marrow stromal cells are being more comprehensively analyzed following the advent of single-cell sequencing [54]. In order to clarify the cellular taxonomy in mouse bone marrow, Scadden’s lab had performed comprehensive single-cell sequencing for overall stroma cell from bone marrow and demonstrated that the bone marrow comprising Lepr-MSCs, pericytes, 2 subtypes of osteogenic lineage cells, 4 subtypes of chondrocytes, 3 subtypes of endothelial cells and 5 subtypes of fibroblasts. Then these cells formed different niches for HSC to maintain normal hematopoiesis by secreting regulatory molecules and accompanied with HSC to maintain the balance of the bone marrow microenvironment [55]. However, once the balance in bone marrow is disrupted, the microenvironment will be patterned. Tikhonova et al. revealed significantly transcriptional remodeling of these niche elements under stressful conditions [56]. Besides, leukemia was found to induce vasculature remodeling with decreased osteocalcin (*Bglap*) serum levels [57, 58]. We elaborate on the cell types in the bone marrow as follows.

A) Endothelial cells in bone marrow

Bone is a highly vascularized tissue, and the inner surface of the blood vessels has a single layer of endothelial cells. Most of the blood vessels in the bone marrow are sinusoidal capillaries, while arteries belong to the minorities in the bone marrow vessels [59]. It has been shown that angiogenesis and osteogenesis are coupled in the bone. Located in the epiphysis and endosteum of postnatal long bones, H-type endothelium labeled by CD31^hi^/Emcn^hi^ mediated angiogenesis in bone and provides niche signals to a large number of surrounding MPs, promoting the osteogenic capacity of MPs [60]. Tikhonova et al. revealed two types of endothelial cells residing in the bone marrow with single-cell sequencing by employing VEcad-cre; LoxP-tdTomato mice to label vasculature cells. One group of VEcad^+^ cells express arterial-associated genes (*Ly6a*, *Icam2*, *Podxl*) and the other one showed sinusoidal signature with high expression of *Stab2*, *Flt4* and *Icam1* [56]. It was very intriguing that Delta-like (Dll)1 and Dll4, two Notch ligands, were specifically appeared in the VEcad^+^ cells. Previous studies have demonstrated that endothelium-specific disruption of notch signaling resulted in decreased osteogenesis and chondrogenesis, as well as trabecular loss and reduced bone mass [61]. Likewise, Scadden’s single-cell data from bone marrow had shown two different endothelial subsets, sinusoidal endothelial cells (Vegfr3^+^Sca1^−^) and arteriolar/capillary endothelial cells (Vegfr3^−^Sca1^+^ or Vwf^+^). Both two types of endothelial cells expressed endothelial-related markers including *Cd34* and *Emcn*. *Cxcl12* and *Kitl* are highest in arteriolar endothelial cells, hence it is likely that arterial endothelial cells act as niche cells that regulate and interact with HSCs [55, 62].

B) Pericytes and perivascular stromal cells in bone marrow

Pericytes are multi-functional perivascular cells (mural cells) that wrap around the endothelial cells locating inside of capillaries and venules, with round nuclei distinguished from endothelial cells which hold flat elongated nucleus. LepR^+^ cells could represent a majority of pericytes and serve as a niche for hematopoietic progenitors [63]. There is accumulating evidence that LepR marks adult BMMPs around sinusoids and arterioles [8]. LepR^+^ cells highly express *Lepr*, *Adipoq*, and MP and SSC markers (*Nt5e*, *Vcam1*, *Eng*, *Grem1*) and therefore possess pre-adipocytic and stem/progenitor-like features [55]. Single-cell data has illustrated that LepR^+^ compartment was divided into 4 pericyte subtypes, respectively representing two differentiation branches: adipogenic and osteogenic directions [55, 56]. Adipo-primed LepR^+^ cells coated sinusoidal capillaries, expressing adipogenesis-associated markers and pro-hematopoietic factors. Among them, a subset of them displayed a stable pro-adipogenic feature. Meanwhile, osteo-primed LepR^+^ cells mainly located in the trabecular bone, highly expressing osteogenic lineage genes (*Wif1*, *Ibsp*, *Spp1*, *Sp7* and *Alpl*) [55, 56]. Furthermore, the majority of bone marrow perivascular MPs with reticular morphology surrounding sinusoids and arterioles express *Cxcl12*, termed Cxcl12-CAR cells, which present as MP-like cells and abundantly express adipocyte-related genes [64]. CAR cells can be separated into Adipo- and Osteo-CAR [65]. The former exhibits a high transcriptome similarity to LepR-Cre cells, expressing high levels of *Lepr*, *Adipoq* and *Lpl* [65, 66]. The latter expresses higher levels of *Sp7*, *Bglap*, and lower levels of *Lepr* [65]. Of note, according to lineage tracing data, the CAR cells become osteoblasts in adulthood [67]. Osteo- and Adipo-CAR are localized on arteriolar and sinusoidal surfaces, respectively, and support the fate determination of immune cells and myeloid lineages by secreting key cytokines, thereby establishing perivascular micro-niches [65]. Additionally, a recent study discovered marrow adipogenic lineage precursors (MALPs) were specifically labeled by *Cxcl12*, *Lepr*, and *Grem1*, which existed copiously as stromal cells and pericytes that constituted a ubiquitous three-dimensional network in bone marrow [68]. MALPs are unable to proliferate as typical MPs, resembling the later differentiation stages of MPs, similar to adipocyte stem cells [68].

Pericytes probably comprise two main types: (1) Osteogenic pericytes located in arterioles give rise to osteogenic lineage cells like Osteo-CAR cells; (2) Adipogenic pericytes located in sinusoidal give rise to adipogenic lineage cells. Adipogenic pericytes should be part of adipose-derived stem cells that have the potential to transdifferentiation into MPs which then further into osteogenic lineage cells. So even the adipogenic pericytes have some MPs properties.

C) Osteogenic lineage cells in bone marrow

As mentioned before, single-cell data had already shown one subset of the Lepr labeled mesenchymal stromal cells highly expressed *Sp7*, *Alpl*, as well as *Runx2*, suggesting differentiation toward the osteogenic lineage. Furthermore, markers of osteogenic differentiation (*Runx2* and *Sp7*) and mature osteoblasts (*Bglap*) were expressed in osteogenic lineage cells cluster 1 and 2. Specifically, the cells in osteogenic lineage cells cluster 1 included state from transitional early osteoprogenitor cells transformed from Lepr-MP to late osteoprogenitors, and finally to mature osteoblasts. The osteogenic lineage cells cluster 2 included osteoprogenitors, osteoblasts and osteo-chondrocytes [55]. Meanwhile, Aifantis’s single-cell data had displayed three osteogenic lineage subpopulations labeled by *Col1*. The first type of Col1^+^ cells expressed high levels of *Ostn*, *Angptl2*, *Edil3* and *Mmp14*. The second ones were abundant in osteogenic genes and chondrocyte markers such as *Col10a1*, which indicated cells undergoing osteogenic transdifferentiation. The third ones represented mature osteoblasts by expressing highest level of *Col2.3* (*Col1a1*) and osteogenesis-related markers [56].

D) Fibroblasts in bone marrow

Scadden’s single cell data indicated that 5 distinct clusters highly expressing fibroblasts gene (*Fn1*, *S100a4*, *Dcn*, *Sema3c*) were divided into two types in bone marrows. The first one is MSC-like who expressed progenitor and MP markers (*Cd34*, *Ly6a*, *Pdgfra*, *Thy1*, and *Cd44*). The other one related to tendon/ligament cells by expressing *Sox9* and *Scx* [55]. MP has a fibroblast-like morphology and some of its surface antigens are expressed at similar levels in MP as in fibroblasts, making the two indistinguishable in bone marrow [69]. Moreover, MPs can generate fibroblasts, and BMMPs are an important source of fibroblasts in the tumor microenvironment [70, 71]. The genes *Dcn* [72], *S100a4* [73, 74], and *Fn1* [75], which are considered fibroblast markers, are as well expressed in MPs, therefore the fibroblast subpopulation marked here may be another subpopulation of MPs.

#### MPs residing in the bone marrow, endosteum, and chondro-osseous junction of metaphysis

At the terminal parts of the long bones are trabecular-rich metaphyseal, regarding as the junction between the bone marrow and the growth plate. The endosteum of long bones wrap around the inner surface of the bone marrow cavity and the surface of the trabeculae of cancellous bone, consists of a thin layer of bone-lining cells and osteoblasts, and contains osteoprogenitor cells [76]. During fetal development, immediately following the initiation of bone collar formation, bone marrow and trabecular bone occupy the center of the cartilage template and create a highly vascularized environment that is considered to host abundant MPs (Fig. 2). The metaphyseal marrow cavity and endosteum may serve as a habitat for MPs in adulthood [77].

It has been shown that BMMPs located near the bone marrow and endosteum of metaphysis behave differently from those in the epiphyseal bone marrow and possess functionally distinct profiles. Metaphysis MPs express reduced cell cycle inhibitors, display high proliferative capacity, metabolic and immunosuppressive activity compared to BMMP in the epiphysis, meanwhile, metaphysis MPs are associated with bone formation as well [78]. Trajectory analysis and fate mapping suggest that PDGFRα^+^β^+^ BMMPs in the postnatal metaphysis can give rise to osteoblast lineage cells, LepR^+^ reticular cells and diaphysis MPs [77]. Intriguingly, metaphyseal BMMPs and diaphyseal BMMPs simultaneously maintain plasticity and the potential for interconversion in respond to injury, aging or disease [77]. The extensive crosstalk between metaphyseal BMMPs and vascular-associated cells and endothelium affects MPs differentiation and bone vasculature. Endothelial cell-derived PDGF-B acts as a key niche factor for bone marrow stromal cell and promotes osteogenic differentiation. Jun-B, which is highly expressed mainly in metaphyseal BMMPs, controls the fate of PDGFRβ^+^ MPs and inhibits adipogenic differentiation. Inactivation of Jun-B in PDGFRβ^+^ MPs resulted in a drastic reduction in H-type vascular columns and arteries [77].

Pdgfrb-CreERT2, Lepr-CreERT2 and Gli1-CreERT2 mice consistently revealed that Pdgfrb^+^, Lepr^+^ and Gli1^+^ cells from postnatal containing MPs firstly emerge in the metaphysis and then proliferate and generate other cell types to populate the bone marrow cavity of the diaphysis [77]. *Pdgfrb*- and *Lepr*-labeled cells from P1-P4 mice and *Gli1*-labeled cells from one-month-old mice are located at the chondro-osseous junction immediately underneath the growth plate [77, 79], revealing the heterogeneity of metaphyseal MPs at different developmental stages. Indeed, since Gli1^+^ cells express a panel of MP markers (*Lepr*, *Vcam1*, *Mcam*, *CD44* and *Pdgfra*), and display tri-lineage differentiation potential, our lab termed them as “metaphyseal mesenchymal progenitors” (MMPs) [79]. Functionally, MMP are indispensable for cancellous bone formation, and support fracture repair by providing chondrocytes and osteoblasts. Hh signaling and β-catenin signaling play vital roles in maintaining MMPs and its osteogenic differentiation [79]. Meanwhile, the MMP population include a chondrocyte-like osteoprogenitor subset with the highest level of Hh target gene and *Igf1r*, serving as a target for teriparatide, a widely prescribed bone anabolic drug [80]. αSMA-labeled progenitor cells, which can generate osteoblasts in vivo and show a three-way differentiation fate of chondrogenesis, osteogenesis, and adipogenesis in vitro induction, are likewise present in the primary cavernous bodies of adult mice [39]. *Sp7* marks at least three different MP subsets with osteogenic potential in fetal, perinatal and adult bone, all inhabiting the endosteum of both spongiosa and cortical bone and providing stromal cells to the diaphyseal bone marrow [20, 81, 82]. However, only perinatal Sp7^+^ cells serve as long-lived self-renewal MPs that contribute to osteolineage cells in the long term and also are precursors of Nes^+^Lepr^+^ MPs in the adult bone marrow [20]. In addition, *Grem1*, a BMP antagonist, label a subpopulation named osteochondroreticular (OCR) stem cells, regarded as postnatal SSCs. OCR stem cells inhabit at primary spongiosa and osse-chondro junction of long bone metaphysis from perinatal mice. Fate mapping of Grem1-expressing cells demonstrates that, as SSCs, Grem1^+^ cells are capable of osteogenesis and chondrogenesis, and produce reticular stromal cells, but not adipocytes, which are different from any other metaphyseal MPs [7]. However, single cell sequence data from endosteum of long bone reveals that adipocyte precursor, including most of pericyte, were also highly labelled by *Grem1* and can exclusively differentiated into adipocytes [68]. This discrepancy may be due to the different ages of the mice used. As we above mentioned that IHH signaling is controlled by prehypertrophic and hypertrophic zone, SSCs and MPs in the trabecular bone can respond to Hedgehog signaling.

#### Marker sets of MPs in postnatal periosteum

Periosteum is a thin layer of vascularized tissue formed by perichondrial cells that attaches to the outer layers of cortical bone (Fig. 2). Periosteum connect with tendon and muscle and is highly responsive to mechanical stress. A large number of existing studies have shown the existence of MPs in the periosteum, and the activation of which is required for bone regeneration after injury [4, 83, 84]. Above mentioned PDPN^+^CD146^-^CD73^+^CD164^+^ hSSCs identified by Longaker’s lab resides in the periosteum as well, besides growth plate [5].

The MPs in periosteum are heterogeneous populations with two bone formation pathways, endochondral ossification and intramembranous ossification. Markers of periosteal MPs that undergo osteogenesis through endochondral ossification are generally identical with the ones of MPs in other structures of bone. Osteoblast-committed Col1a1^+^ cells, residing in the innermost layer of periosteum which directly attached to the long bone surface [85]. Meanwhile, *Prrx1*, a marker of the mesenchymal lineage in developing limbs, likewise label osteochondral progenitor cells in the periosteum, which are immediately outside of the Col1a1^+^ cells and further away from the cortical bone than Col1a1^+^ cells [85, 86]. The sorted Prrx1^+^ periosteum cells display chondrogenic and osteogenic potential, and overexpressed BMMPs markers such as *PDGFRα*, *Grem1*, *Cxcl12*, *Nestin15* and *NG2*, but not *LepR* [87]. However, Gao et al. identified LepR^+^ MPs and Nestin^+^PDGFRα^+^ MPs from the tibia diaphyseal periosteum of adult mice and young mice, respectively, both of which were endowed with self-renewal and trilineage differentiation ability and even exhibited higher osteogenic and chondrogenic potential than BMMPs [88]. Functionally, they both contribute to cortical bone formation, but contrarily, Nestin^+^PDGFRα^+^ MPs abundant in the periosteum of young mice are primarily devoted to skeletal development, whereas LepR^+^ MPs enriched in the periosteum of adult mice are mainly engaged in the maintenance of bone homeostasis [88]. Compared with periosteal MPs with strong chondrogenic potential mediating intramembranous bone formation, periosteal MPs engaging in intramembranous ossification do not involve in the cartilage formation or hematopoietic recruitment [16]. The population of periosteal MPs marked by cathepsin K (CTSK) are the ones that form bone through intramembranous route [16]. Specifically, THY^-^6C3^-^CD49^low^CD51^low^CD200^+^CD105^−^ cells within the CTSK-mGFP^+^ cells from Ctsk-cre; mTmG reporter mice, are considered as periosteal MPs and named periosteal stem cell (PSC), which exhibit self-renewal and multiple differentiation potential even through successive rounds of transplantation. It was observed that PSC appears in the mesenchymal compartment as early as the onset of skeletal mineralization in E14.5. Furthermore, PSCs retain chondrogenesis ability and can form bone through endochondral ossification in respond to injury.

Unlike BMMPs whose primary role is to form the HSCs niche and regulate bone turnover, periosteal cells are the main cells that respond to injury. During bone formation and repair, MPs in bone marrow and periosteum are marked by different marker genes, such as *Ctsk* in periosteum and *Cxcl12* in bone marrow. MPs in postnatal periosteum display enhanced differentiation and proliferation compared to BMMPs [87], which seems to imply a higher hierarchy of periosteal MPs. Besides, the microenvironment at which these two cell types locating are completely different. In periosteal niche, macrophage lineage cells influence periosteal MPs recruitment and stemness, thus regulating the homeostasis of periosteum and cortical bone, as well as repair processes. By secreting platelet-derived growth factor-BB (PDGF-BB), macrophage-lineage cells induce the expression of periostin that is are necessary to support periosteal cells and control their stemness [87], and recruit MPs to the periosteal surface to regenerate cortical bone [88, 89]. In macrophage-lineage deficient mice, cortical bone is extremely thin and abundant unmineralized trabecular bone is present, suggesting a different regulation mechanism during bone remodeling between MPs in these two types of bone [89].

### Aging alters lineage commitment tendencies and stemness of MPs

**Fig. 3:**
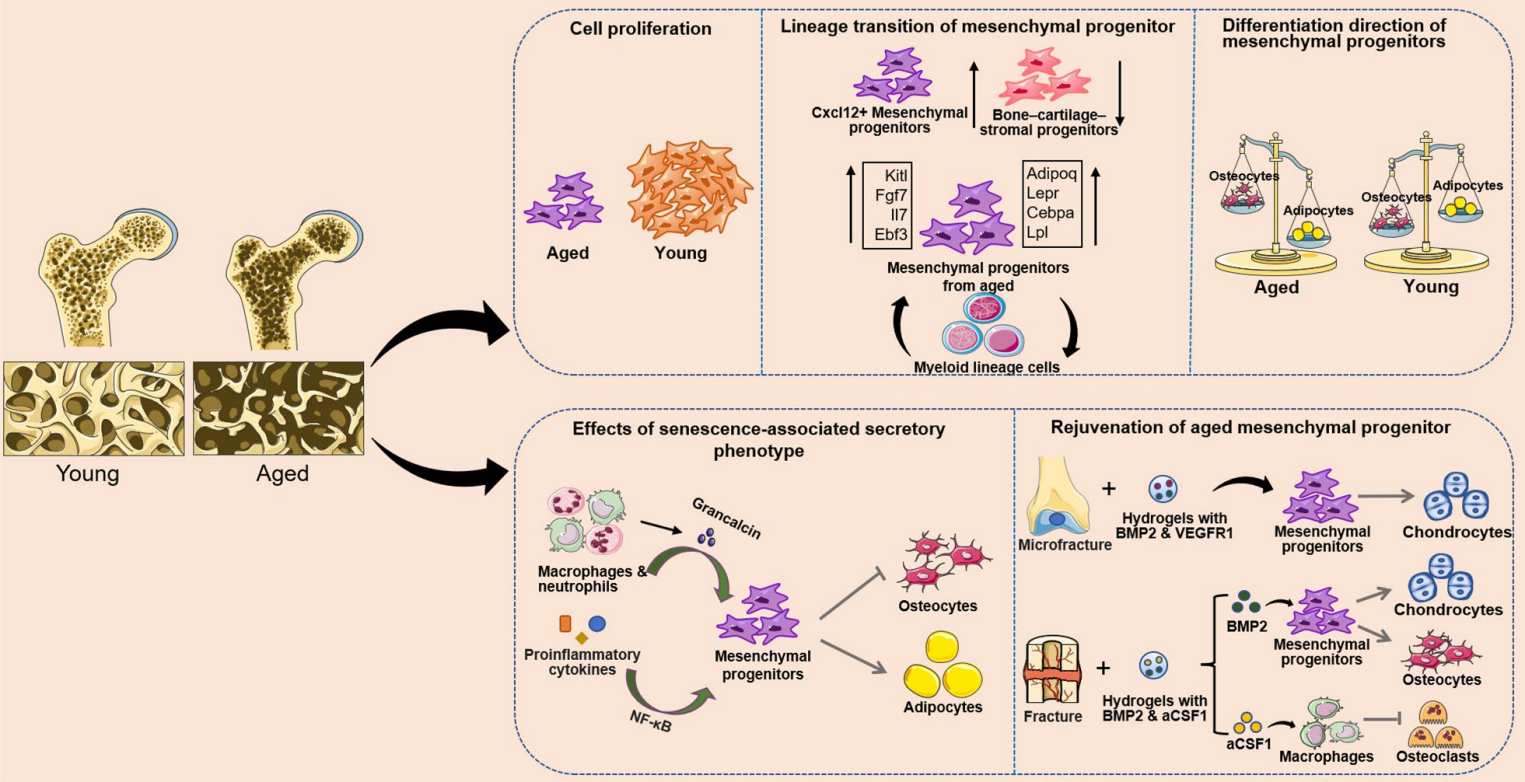
Aging and rejuvenation of mesenchymal progenitor

With aging, the osteogenic capacity of MPs in the bone tissue declines and tend to differentiate into adipocytes, which lead to a corresponding decreased bone mass, making the elderly susceptible to aging-related bone fracture (Fig. 3). Studies had shown that human MPs from older donors retained impaired proliferative and osteogenic capacities compared to those from young donors [90]. In mice, Thomas et al. observed a decrease in the number of total MPs and bone–cartilage–stromal progenitor (BCSPs) [6, 91], and a reduce in the proliferative activity of MPs in the bone tissue of 24-month-old mice versus 2-month-old mice. The downstream output of MP progenies in 24-month-old mice shifted toward myeloid lineage populations and away from stromal cell [91]. In line with this observation, Matthew et al. revealed a significant diminishment in MPs, osteochondral and stromal progenitors in adult mice compared to P3 mice, likewise MPs isolated from adult mice possessed a reduced clonogenic potential in vitro compared to the MPs derived from P3 [92]. Furthermore, the remarkably reduction of Sca-1^+^CD29^+^CD45^−^ endosteal MPs is observed in aged mice, which is responsible for the loss of bone trabeculae. While, parathyroid hormone treatment redeems the decrease of epiphyseal endothelial MPs caused by aging and stimulates bone formation [78].scRNA-seq data demonstrated differences in the marker of MPs between young and aging mice as well as the restricted lineage commitment of aged SSCs [68]. Lepr which is much more highly expressed in MPs of aging mice then in young mice, is referred as marker of adult MPs, not young MPs [8, 88]. Adipocyte markers, such as *Cebpa* and *Lpl*, expressed at higher levels in MPs of aging mice, exhibiting adipocyte drift in aging MPs [68]. In addition, the proportion of BMMP expressing CXCL12 and early B-cell factor 3 (EBF3) in osteogenic lineage cells increases with age as well [64, 67]. Cxcl12^+^ BMMPs retained in the bone marrow space abundantly express adipocyte-associated genes (*Adipoq* and *Lepr*) and multiple cytokines (*Kitl*, *Fgf7*, *Il7*) [64]. While *Ebf3* is specifically expressed in bone marrow CAR-MPs after birth. In the absence of *Ebf3*, most bone marrow CAR cells of aged mice incline to product osteogenic lineages, thus severely impaired HSC niche and osteosclerosis with increased bone are present in aged mice [67]. In contrast to the multiple distinct fate specification programs of young SSCs, aged SSCs are most abundant in the pro-hematopoietic stromal cluster and are absent in chondrogenic cluster and extracellular matrix gene-expressing stromal cluster. Meanwhile, aged SSCs were enriched for genes related to decreased bone formation, increased monocyte and macrophage chemotaxis, and enhanced bone resorption, suggesting that osteogenic potential of the aged SSCs were diminished and skewed toward the pro-medullary axis [91]. Osteocytes, located at the end of osteogenic differentiation, are an essential source of signals regulating myeloid cells. It modulates the development of granulocytes and neutrophils by secreting IL19 [93], and secreting factors that control myeloid cell proliferation and inhibit osteoclasts through Gsα-dependent and non-dependent mechanisms [94]. Notably, osteocyte produces RANKL, a key cytokine for activation of osteoclast differentiation, which regulates osteoclastogenesis and thus control bone resorption [95]. The decrease in osteocytes causes osteoblasts and myeloid cells to acquire senescence-associated secretory phenotype (SASP), which impairs osteogenesis, induces adipogenesis, and promotes osteoclastogenesis, accelerating bone aging [96]. In addition, it has also been shown that MPs and osteogenic lineage cells mediate osteoclast activity. Cyclin-dependent kinase 8 (CDK8) in MPs, controls osteoclastogenesis externally via the STAT1-RANKL axis. Moreover, CDK8 in MPs is associated with senescence-related signals, and its expression is positively correlated with age, thus aging MPs highly support osteoclastogenesis [97]. Specifically, Aged SSC and MPs lose transcriptome diversity to a certain extent [91], increase preference to myeloid and adipogenic lineage, and interact with hematopoietic compartments, which can ultimately promote adipogenesis and even osteoclastogenesis [91].

In addition to cell-autonomous changes in the SSCs and MP, the age-related local microenvironment is described as the major contributor to the shifted lineage commitment and the declined regeneration [91]. Senescent cells with SASP excrete SASP-associated chemokines/cytokines, that not only enhance their own aging response but also expedite senescence of neighboring non-senescent cells through paracrine mechanisms. Recently, Li et al. revealed that during aging in rats and mice, proinflammatory senescent immune cells, including macrophages and neutrophils, accumulated and secreted large amounts of grancalcin in the bone marrow, which are found to inhibit osteogenesis and promotes adipogenesis in bone marrow MPs by binding to plexin-b2 receptors in MPs and partially inactivating its downstream signaling pathways [98]. Based on these findings, they developed a grancalcin-neutralizing antibody that effectively improves bone health in aged mice [98]. Age-related proinflammatory environment through nuclear factor kappa-B (NF-κB) inhibits proliferation and cellular function of skeletal stem/progenitor cell (SSPC) [99, 100]. However, this inflammation-associated decline in SSPC is reversible. Anti-inflammatory treatment of aged mice with sodium salicylate, an inhibitor of NF-κB pathway activation, could suppress aging-induced chronic inflammation and functionally rejuvenate aging SSPC, resulting in increased SSPC numbers and osteogenesis [90].

As an important niche of MP, bone matrix can release various factors and participate in the regulation of MP differentiation. Insulin-like growth factor 1 (IGF-1) and active transforming growth factor-β1 (TGF-β1), are two key osteogenic regulators of bone matrix origin [101, 102]. IGF-1 and TGF-β1 are involved in maintaining bone turnover by mediating mTOR and SMAD signaling pathways to recruit MPs and induce MP to differentiate into osteoblasts, respectively [103, 104]. Aging-related osteoporosis was significantly negatively correlated with bone content of IGF-1 and TGF-β1 [103, 104]. Experiments have shown that injection of IGF-1 + IGF binding protein 3 increased the concentration of IGF-1 in bone matrix and promoted new bone formation in aged rats [104]. TGFβ-1 is released by osteoclasts during the resorption of bone matrix. It stimulates MP to migrate to remodeling site, and enhances osteogenesis of MP, thereby maintaining the balance between bone formation and resorption. TGFβ-1 simultaneously is a key molecule in cartilage formation, which stabilize SOX9 through SMAD or p38 pathway to induce the differentiation of MP into chondrocytes [105, 106]. In contrast, bone matrix extracellular vesicles secreted by osteocytes attached to aged bone matrix favor adipogenesis with MP [107]. However, the use of the alendronate inhibits aged bone matrix extracellular vesicle release, and attenuates the bone-fat imbalance induced by aging and ovariectomy [107].

Progressive decrease in the amount of SSC and chondrogenesis in aging joints is associated with the development of osteoarthritis. However, microfracture surgery stimulates the cartilage surface of adult mouse limb joints to trigger local expansion of SSC and tilt differentiation of microfracture-activated SSC toward articular cartilage in the presence of BMP2 and soluble VEGFR1, providing new ideas for the reconstruction of articular cartilage in Osteoarthritis [92]. Similarly, to address the slow fracture healing caused by aging, Thomas et al. found that combined treatment with low doses of a CSF1 antagonist (aCSF1) and BMP2 increased the percentage of osteoblast-lineage cells in calluses of 24-month-old mice in which SSCs exhibited youthful colony formation and osteogenic capacity, while aCSF1 inhibited osteoclast maturation, thereby reducing bone resorption [91]. The combination of the two aspects contributed to the mechanical strength of the healed aged femur matching that of young bone and the regenerative capacity of the aged bone was restored to youthful levels. Conversely, 24-month-old mice treated with PBS showed an abundance of myeloid-derived immune cells in the bone calluses [91].

## MPs respond to skeletal injury

### Bone fracture

After a fracture, MPs are the primary responsive cells in the injured area that replenish the osteoblasts for regenerating bone tissue and eventually restore the original structure and function at the fracture site. The majority of MPs that contributes to bone regeneration are of periosteal and bone marrow origin [84]. Although both these two types of MPs derived from the same embryonic stem cell lineage, they make distinct contributions to bone repair (Table 3), and in addition, periosteal MPs possess a higher bone regenerative potential and a stronger response to bone fractures than bone marrow-derived MPs [108, 109].

**Table 3:** MP population in long bones in response to injury

Sources of MPs in response to repair	Marker	Function	References
Periosteum	Gli1^+^	The repair of bicortical fractures	[109]
Periosteum	Mx1^+^αSMA^+^	The formation of new periosteum	[20, 81, 82, 108]
Periosteum	Sox9^+^	Migrating toward fracture site as early response	[112]
Periosteum	CTSK^+^THY^−^6C3^−^CD49^low^CD51^low^ CD200^+^CD105^−^	Providing chondrocytes for fracture callus	[16]
Bone marrow	LepR^+^Adipoq^+^	The repair of drill-hole injuries	[109]
Bone marrow	CD45^+^TER119^−^Tie^−^AlphaV^+^Thy^−^6C3^−^CD105^+^	Expanding shortly after injury before formation	[6]
Bone marrow	Cxcl12^+^	The regeneration of cortical bone	[64]
Bone marrow	Gli1^+^	The formation of bone and cartilage after fractures	[79]
Bone marrow	LepR^+^	The formation of soft callus	[8]
Bone marrow	Grem1^+^	Differentiating into osteochondral fracture callus	[7]
Bone marrow and periosteum	αSMA^+^	The formation of fracture callus	[39, 111]
Perivascularization of the periosteum and endosteum	Prx1^+^	Contributing to the callus	[116]
Endosteum	CXCL12^+^BMP2^+^	Transfering to pericytes-osteoblasts-osteocytes fate to form new bone	[117]

Specifically, periosteal MPs was demonstrated to display a greater potential for colony-forming and differentiation [87]. According to Duchamp de Lageneste’s research, periosteal MPs, augmented by strong expression of extracellular matrix gene (e.g. *Periostin*), can largely reconstitute new periosteum after injury, and notably the new generated periosteal cells reconstitute periosteum again in the next injury cycle [87]. Lineage tracing data showed that αSMA labeled resident periosteal MPs are activated in early healing time point [110]. αSMA^+^ MPs invaded into fracture site 2 days after injury, and their progenies robustly expanded and differentiate toward osteogenic and chondrogenic lineages 6 days later [39, 111]. Subsequently, postnatal Mx1^+^αSMA^+^ periosteal MPs were further characterized, which specifically expressed CCL5 receptors, CCR3 and CCR5, migrated to the injury site mediated by CCL5 and replenish osteoblasts as well as chondrocytes for both cortical bone regeneration and new periosteum formation [108]. In addition to labeling MMP in the chondro-osseous junction of young mice, Gli1 likewise labeled SSCs in the periosteum of adult mice that were primarily responsible for repairing bicortical fractures. During endochondral osteogenesis in fracture repair, chondrocytes in the soft callus and osteoblasts in the hard callus were largely derived from Gli1^+^ cells, while periosteal SSCs were major contributors to newly generated cortical bone at the healing site. However, osteoblasts in newly formed new trabecular bone within the bone marrow at the fracture site were mostly derived from Adiponectin^+^ BM cells [109]. A subpopulation of MPs in periosteum that likewise contribute to healing in the early stages of fracture repair are Sox9^+^ cells. Fate mapping indicated that resident or de novo formed Sox9^+^ periosteal MPs in adult long bones migrated toward the injury site and differentiated into chondrocytes and osteoblasts as early as 3 days post fracture [112]. Recently, Debnath et al. revealed that periosteal MPs expanded in response to injury and were inclined to osteogenic differentiation [16]. CTSK-mGFP^+^ periosteal MPs gave rise to approximately half of the chondrocytes in the soft callus. Mechanistically, the ablation of Osx in Ctsk-cre^+^ cells resulted in significantly impaired fracture healing, defective mineralization, and reduced bone volume and increased cartilage in callus of mice [16], which suggested the indispensable role of Osx in bone fracture healing.

Although inferior to periosteal MPs in injury repair, BMMPs possess a greater regenerative capacity than BMMPs from uninjured bone when activated by injury-induced microenvironment changes. It was shown that BCSPs (CD45^−^TER119^−^Tie2^−^AlphaV^+^Thy^−^6C3^−^CD105^+^), a subpopulation of MPs which are referred as progenitor for chondrocytes osteogenic cells and stromal cells, can be characterized by CD49f after injury, i.e., CD45^+^TER119^−^Tie^−^AlphaV^+^Thy^−^6C3^−^CD105^+^ [6, 113]. Compared to uninjured-BCSPs, injury-induced CD49f^+^ BCSPs are active during the acute repair period, are endowed with enhanced proliferative capacity and osteogenic potential, and maintain a perinatal phenotype in adult skeletons [6]. Matsushita et al. demonstrated a significant increase in Cxcl12-creER^+^ BMMPs CFU-Fs ex vivo after bone marrow ablation [64]. Similarly, single-cell data illustrated that Cxcl12-creER^+^ BMMPs after bone marrow ablation tended to shift toward preosteoblasts and osteoblasts, with upregulation of osteoblast signature genes in reticular and preosteoblast clusters [64]. In addition to marrow ablation, quiescent Cxcl12-creER^+^ BMMPs can be recruited to injured cortical bone and produce osteoblasts. In complete fractures, Cxcl12-creER^+^ BMMPs also produce chondrocytes to promote endochondral ossification and bone remodeling [64]. Of course, the contribution of BMMPs to fractures should not be underestimated. Adiponectin- and Lepr-labeled bone marrow SSCs predominate in the repair of drill injuries [109]. Gli1^+^ MPs in the chondro-osseous junction are regard as skeletal stem/progenitor reservoir for bone and cartilage formation after femur fractures, producing approximately 63% aggrecan^+^ chondrocytes and 50% Ocn^+^ osteoblasts, respectively, in bony callus after 10 days of fracture [79]. NFATc1^+^ SSCs, which largely overlap with Gli1^+^ MP in the metaphyseal region, are involved in early callus formation, and their progeny osteoblasts are abundant in newly mineralized bone tissue [114]. Moreover, even though LepR^+^ cells make a minimal contribution to cartilage formation during development and LepR^+^ BMMPs are quiescent in the bone marrow of adulthood, fracture drives the differentiation of LepR^+^ BMMPs towards osteochondral lineage, and their progeny chondrocytes accounts for approximately 46% of Aggrecan^+^ chondrocytes in the soft callus 2 weeks after fracture [8]. Single-cell data exhibited an increase in the proportion of osteogenic lineage subpopulations in the LepR-Cre^+^ BMMP after fracture, an upregulation of secreted bone matrix factors such as *Col1a1*, *Col1a2*, *Sparc*, and *Bglap* in the this subpopulation, as well as an expansion of ossification gene (*Bglap*)-enriched subpopulation in the adipogenesis lineage [115]. In addition Hoxb2 and Npdc1 regulons, as potential regulators of osteochondrogenic differentiation, are upregulated after fracture [115]. The Grem1^+^ osteochondroreticular BMMPs identified by Worthley et al. produced only about 28% of the Col1^+^ osteoblasts and about 14% of the Sox9^+^ chondrocytes in the callus 6–8 weeks after fracture. In contrast, other BMMPs and Mx1^+^ periosteal MPs contributed much more (over 80%) to osteoblasts [7].

The endosteum is one of the habitats of fracture response MPs as well. Perivascular Prx1^+^ cells around periosteum and endosteal are responding to injury, leading to an upregulation of BMP2 in early differentiation and then a downregulation of *Prx1* via CXCL12 signaling, which facilitate MPs to osteochondrogenesis [116]. There is evidence for a CXCL12^+^BMP2^+^endothelial cell-specific response in the early stages of fracture repair. BMP2 downregulates CXCL12 and CXCL12 support factors in endothelial cells may be a critical switch from perivascular cell to osteoblast function, leading to osteoblast differentiation and fracture healing. This response decreases progressively with the progression of healing and is not present in unfractured bone [117].

Other tissues besides bone, such as vascular tissue [118] and adjacent soft tissue [119], may also contribute MPs to fracture repair. There is evidence that vascular pericytes exhibit a trilineage differentiation potential with osteogenic, chondrogenic, and adipogenic abilities [118]. Kumagai et al. using parabiotic murine model illustrate that circulatory-derived osteogenic stem/progenitor cells are mobilized to establish soft callus and promote osteogenesis during the first two weeks of fracture healing [118, 120].

### Osteoporosis

Bone homeostasis is a dynamic equilibrium maintained by a combination of osteoclast-dominated bone resorption and osteoblast-dominated bone formation. Osteoporosis is caused by an imbalance between osteoclasts and osteoblasts, and an abnormal allocation of the MP lineage is one of the important factors triggering osteoporosis. MP differentiation in long bones of patients with osteoporosis is shifted toward adipogenesis at the expense of osteogenesis, resulting in a large proportion of marrow adipose tissue in the marrow cavity [121, 122]. Parathyroid hormone is the main hormone that regulates calcium and phosphorus metabolism and maintains calcium homeostasis in the body, while it acts directly on osteoblasts and affects bone homeostasis. Knockdown of the PTH/PTHrP receptor (PTH1R) in Prx1-Cre-targeted MP causes MP to express classical adipogenic markers (*Fabp4*, *Adipo*, *Perilipin*, etc.) and to differentiate uncontrollably into bone marrow adipocytes, implying diminished osteogenesis; and the subsequent generation of preadipocytes secretes RANKL. The combination of these two effects leads to the accumulation of bone marrow adipose tissue and reduction in bone mineral density, progressing to osteoporosis [122]. Peroxisome-proliferator-activated receptor γ coactivator (PGC-1α), which acts as an osteogenic protector, promotes osteogenesis and impairs adipogenesis. PGC-1α and its coactivator nuclear respiratory factor-2 (NRF2) bind to the promoter of TAZ (transcriptional co-activator with PDZ-binding motif) to control TAZ expression and subsequent osteogenic gene expression. PGC-1α deletion allows MP to promote adipogenesis in mice MP at the cost of osteogenesis. Prx1-cre; Pgc1a^f/f^ and LepR-cre; Pgc1a^f/f^ mouse exhibit enhanced bone loss, osteogenesis inhibition and bone marrow adipose tissue accumulation after ovariectomy (OVX) [123]. It has been shown that autophagy regulates osteoblast differentiation and mineralization [124], and chondrocyte differentiation [125], which has implications during osteoporotic bone loss. OPTN is an autophagic receptor that plays a pivotal role in selective autophagy. The selective autophagic substrate Fatty Acid Binding Protein 3 (FABP3) accumulates in Optn^−/−^ MP, with enrichment of senescence markers (*p15*, *p16*, *p21*), decreased osteogenic activity and increased adipogenic activity. In contrast, overexpression of Optn or inhibition of Fabp3 in aged mice attenuated bone loss. In addition to FABP3, Deptor is another known proponent of adipogenesis in MP. It is upregulated in adipogenic induction and downregulated in osteogenic induction of MP in vitro; it is upregulated in MP from ovariectomy-induced osteoporotic mice, in vivo. In contrast, targeted knockdown of Deptor in MP with Prrx1-Cre in mice with osteoporosis promote MP osteogenesis and inhibits adipogenesis to alleviate bone loss and bone marrow fat accumulation after OVX [126].

### Osteoarthritis

Osteoarthritis is a chronic degenerative disease characterized by progressive destruction of articular cartilage, synovitis, subchondral bone remodeling, and osteophytes formation [127]. Progenitor cells resembling MP are present in articular cartilage that most research groups have defined as chondrogenic progenitor cells (CPCs) or cartilage-derived stem/progenitor cells (CSPCs), but there is an absence of a universally accepted marker set. Several studies have shown that CPCs or CSPCs from articular cartilage possess multilineage potential and their osteogenic and adipogenic capacity is comparable to that of bone marrow-derived MPs [128–131]. In addition, as reparative cells maintaining cartilage homeostasis, they are endowed with the ability to respond to injury and migration and are involved in the entire phase of osteoarthritis. [127, 132, 133]. In early osteoarthritis, most CPCs are located in cartilage, in or near the cartilage fissure and express Notch-1, STRO-1 and VCAM-1 [127, 132]. Runx2 is down-regulated in CPCs during the later stages of osteoarthritis, displaying high chondrogenic and migratory potential, while this population of CPCs located in the repair region may originally reside in articular cartilage or migrate from the bone marrow via vascularized cartilage [132]. The bone tissue beneath the cartilage is referred to as subchondral bone. Overactive TGF-β in the subchondral bone of osteoarthritis recruits MPs and osteoprogenitor cells, causing abnormal osteogenesis and angiogenesis, thus promoting osteoarthritis progression [134]. Sun et al. found that intermittent parathyroid hormone treatment could maintain bone remodeling homeostasis and improve microarchitecture of subchondral bone in osteoarthritis mice, via interfering with TGF-β signaling to attenuate the recruitment and expansion of Nestin^+^ and Osterix^+^ MP in bone marrow [135]. Osteophyte, a common pathological outgrowth of cartilage and bone in osteoarthritis, originate from MPs expressing Pdgfrα in the periosteum and synovium. PDGFRα^+^GDF5.^−^ MPs are activated at the periosteal-synovial junction near the articular cartilage with osteoarthritis. Sox9-expressing MPs in the periosteum give rise to hybrid skeletal cells expressing *Col2a1*, which co-express osteoblasts and hypertrophic chondrocyte markers (*Ocn*, *Col1a1*, *Spp1*, *Col10a1*), form temporary cartilage templates, and remodel into bone. The progenies of Prg4-expressing progenitor cells in the synovial lining supply the cartilage cap to the osteophyte [136].

### Rheumatoid arthritis

Rheumatoid arthritis is a chronic systemic autoimmune disease in which the disruption of bone remodeling homeostasis is dominated by an excessive immune response. Osteoclasts appear to be highly crosstalked with the immune system, which increases osteoclastogenesis via stimulating preosteoclasts in multiple manners, of which RANKL is a major mediator [137]. RANKL expression is activated in inflammation-activated synovial fibroblasts, T cells and B cells [138–141]. T-helper 17 (T_h_17) cells from CD4^+^ T secrete IL-17, IL-21, and IL-22, which stimulate the expression of RANKL and matrix metalloproteinases in synovial fibroblasts, induce osteoclastogenesis and increase the proliferation of neutrophils and macrophages, leading to further enrichment of pro-inflammatory cytokines and chemokines. Desialylation of IgG by T_h_17 cells enhance the ability of IgG immune complexes to enhance osteoclastogenesis through Fc receptors [142]. In addition, pro-inflammatory cytokines target preosteoclasts directly to augment signaling downstream of RANK and to upregulate the expression of RANK co-stimulatory receptors [143–145]. In addition to osteoclast activation, the inflammatory response suppress osteogenesis as well. TNF, a key inflammatory mediator in rheumatoid arthritis, inhibits osteogenesis by suppressing the expression of osteogenic genes in MP (*Col1*, *Bglap*, *Runx2*), upregulating Wnt signaling inhibitors in synovial fibroblasts (DKK1, SOST, SFRP) [146, 147], and leading to the generation of BMP3, an endogenous BMP inhibitor, by osteoblasts [148]. Macrophages, T cells, and B cells in rheumatoid arthritis are all major producers of TNF [149]. In addition synovial-derived IL-6, macrophage-derived IL-1, and subchondral bone marrow B-cell-derived CCL3 lead to osteogenesis and chondrogenesis inhibition [150]. Among these, TNF-α, IL-6 and IL-1 are negative regulators of cartilage and bone, inducing the formation of matrix metalloproteinases and prostaglandins and blocking the production of Collagen type II [151]. CCL3 or TNF down-regulate osteogenesis and chondrogenesis by activating extracellular signal-regulated kinase and NF-κB signaling pathways [152].

## Discussion

The so-called mesenchymal stem cells (MSCs) population has always been a very controversial one. Although they have been given the term of “stem cells”, the proliferative capacity and the potential for trilineage differentiation in vitro possessed by MSCs cannot be equated with the capacity of stem cells to self-renewal and form different functional tissues in vivo. Therefore, mesenchymal progenitor is considered a name more consistent with the identity of this population in this review. Notably, MPs are very heterogeneous, as they are distinct subpopulation at different stages and locations, reflecting different skeletal tissue fates through various cell surface marker profiles. Specific markers from certain progenitor in this subpopulation might not to apply to another subgroup. Furthermore, the study of MP is complicated by distinct strategies for detection, isolation, and differentiation potential and functional assessment, coupled with a complex microenvironment and unknown heterogeneity, creating multiple barriers to truly characterize MPs. The concept of SSCs was first proposed in 2015, which is endowed with more restricted differentiation fate than MPs, only towards osteogenesis and chondrogenesis, lacking the adipogenic potential in MPs [4]. Early-stage SSCs may have similar properties to MPs, such as CFU-F formation and maintenance of multilineage differentiation, however, late-stage SSCs lose some of the properties of MPs, such as the lack of CFU formation ability, but still hold the potential for multilineage differentiation. A developmental hierarchy of lineage restricted progenitors might throughout the whole skeletogenesis as occurs in hematopoiesis. Although it is difficult to explore an universal set of surface markers to label all MPs in a variety of skeletal tissues currently, just like almost any kind of cells could reprogram into very beginning stem cells (iPS cells) through combination of several Yamalaka factors [153, 154], it might also exist several key factors that gradually determine the SSCs fate.

Elucidating MPs is critical for understanding skeletal injury response. Although great progress in identifying the MPs have been made, it is only the most preliminary comprehension of their nature. MPs originate from a wide range of sources, and many subpopulations which respond differently to injury distribute in different structures in long bone. In addition, distinct sites of injury in long bones induce two different bone regeneration pathways. Periosteal defects repair via endochondral bone formation, while bone marrow defects heal via intramembranous bone formation, each of which is dominated by a different MP subpopulation [84]. Intramembranous ossification-mediated repair is commonly seen in bone marrow injuries and metaphyseal fractures, and MPs in the endosteum are the main responsible subgroups. It is characterized by direct differentiation of MP into osteoblasts, which in turn deposit mineralized extracellular matrix, and a shorter repair time than endochondral ossification. The endochondral ossification pathway is complex and is broadly divided into inflammation, the formation of soft and hard bone callus, and bone remodeling, which commonly occurs in diaphyseal defects. It likewise involves the interaction of multiple cell types, such as the crosstalk between MP and CD169^+^ macrophages [155, 156]. Most of the MPs required for endochondral ossification are recruited from bone marrow and periosteum, and the periosteum is the mainstay of chondrocyte production in callus [84, 110]. In challenging healing environments, such as the presence of inhibitors of skeletal healing (aging, diabetes, obesity, hormonal deficiencies, etc.) or large segmental osseous defects, bone formation and bone remodeling are disturbed or deficient. In this case, pharmacological intervention and bone tissue engineering are necessary [157–159]. The selection of targeted subpopulations of MPs as drug targets or stem cell material in tissue engineering for local or global bone regeneration control and bone repair enhancement, depending on the situation, may be the key to optimize and integrate bone healing therapies in the future. For example, teriparatide [hPTH (1–34)] is approved to treat osteoporosis. Treatment of teriparatide greatly increased the bone mass by elevating the rate of bone production rate [160], in which might through upregulation of Wnt/β-catenin signaling [161]. Lineage tracing experiment indicated that besides direct effect of teriparatide on mature osteoblasts and osteocytes, it stimulated the proliferation and osteoblast differentiation of Gli1^+^ MPs and suppressed apoptosis of Sox9^+^ MPs as well, resulting in bone mass increase [17, 80].

## Conclusion

The development of long bones is initiated by the ectomesenchyme condensation, during which cells in the central part of the condensation differentiate into chondrocytes and secrete cartilage matrix, while other surrounding cells form the perichondrium. These chondrocytes undergo endochondral ossification and begin to form primary and secondary osteogenic centers with the invasion of blood vessels, initiating the journey to osteogenesis. MPs gradually differentiate from the same mesenchymal cells over the course of development to form a complex lineage. Vascular invasion and cell migration likewise introduce MPs of other embryonic origin into the long bones. All of the above MPs form a large and redundant lineage, present in almost all parts of the long bone including the marrow cavity, endosteum, growth plate, and periosteum, as a homogeneous and heterogeneous coexisting population. The homogeneity is reflected in the fact that the same marker can label several different MP subpopulations, some of which are even simultaneously markers for other cell types, such as pericytes and osteoblasts. And all these subpopulations possess the capability to proliferate and produce osteoblasts, chondrocytes, or adipocytes. Whereas the heterogeneity between MPs is high, both in the temporal and spatial dimensions. First of all, MPs with diverse markers exist in long bones at different stages of growth and development and perform dissimilar functions. For example, the adult-specific BMMP marker LepR and the MP population in older bone marrow skewed toward the myeloid and adipogenic lineage. Secondly, MPs from various spatial sites of the long bones are distinct in terms of markers and function. Periosteal MPs exhibit a greater capacity for injury repair than BMMPs, while BMMP not only maintain bone turnover but also act as niche cells of HSC producing various cytokines and growth factors to support hematopoiesis. Furthermore, MPs are endowed with high-level plasticity and create a sophisticated crosstalk network with niche cells, maintaining a dynamic balance under the precise regulation of multiple signals. Pro-inflammatory factors are important regulators of MP and significantly influence MP proliferation and differentiation. Pro-inflammatory factors are important regulators of MP and significantly influence MP proliferation and differentiation. In conditions such as ageing, osteoporosis and arthritis, skewed differentiation of MP lineage and enhanced osteoclasts are achieved through interactions with immune cells and the induction of inflammatory signals in microenvironment.

## Data Availability

Not applicable.

## Abbreviations

MPs: Mesenchymal progenitors
SSCs: Skeletal stem cells
HSC: Hematopoietic stem cells
BMP: Bone morphogenetic protein
FGF: Fibroblast growth factor
CAR cells: Abundant reticular cells
Cxcl12: Chemokine (C-X-C motif) ligand 12
POC: Primary ossification center
Ihh: Indian hedgehog
PTHrP/PTHLH: Parathyroid hormone-related protein
MMP-13: Matrix metalloproteinase 13
VEGF: Vascular endothelial growth factor
hSSCs: Human skeletal stem cells
BMMPs: Bone marrow mesenchymal progenitors
SOC: Secondary ossification center
mTORC1: Mammalian target of rapamycin complex 1
Dll: Delta-like
MMPs: Metaphyseal mesenchymal progenitors
BCSPs: Bone, cartilage and stromal progenitors
MALPs: Marrow adipogenic lineage precursors
BMP: Bone morphogenetic protein
OCR: Osteochondroreticular
CTSK: Cathepsin K
PSC: Periosteal stem cell
PDGF-BB: Platelet-derived growth factor-BB
CDK8: Cyclin-dependent kinase 8
NF-κB: Nuclear factor kappa-B
aCSF1: A CSF1 antagonist
IGF-1: Insulin-like growth factor 1 TGF-β1: transforming growth factor-β1
SASP: Senescence-associated secretory phenotype
SSPC: Skeletal stem/progenitor cell
PTH1R: PTH/PTHrP receptor
PGC-1α: Peroxisome-proliferator-activated receptor γ coactivator
NRF2: Nuclear respiratory factor-2
OVX: Ovariectomy
FABP3: Fatty acid binding protein 3
CPCs: Chondrogenic progenitor cells
CSPCs: Cartilage-derived stem/progenitor cells
T_h_17: T-helper 17
MSCs: Mesenchymal stem cells
